# Evaluation of a Novel Technique for a 3D-Printed Temporal Bone Analog for Mastoidectomy Training

**DOI:** 10.7759/cureus.112098

**Published:** 2026-07-05

**Authors:** Emily J Bacalao, Gabriela Heslop, Jeremy Yang, Lawrence Smith, Robert Mac Curdy, Emily Cooper, Brian W Herrmann

**Affiliations:** 1 Otolaryngology - Head and Neck Surgery, School of Medicine, University of Colorado Anschutz, Aurora, USA; 2 Pediatric Otolaryngology, Cincinnati Children's Hospital Medical Center, Cincinnati, USA; 3 Pediatric Otolaryngology, Rady Children's Hospital San Diego, San Diego, USA; 4 Mechanical Engineering, University of Colorado, Boulder, USA; 5 Biostatistics, School of Medicine, University of Colorado Anschutz, Aurora, USA; 6 Pediatric Otolaryngology, Children's Hospital Colorado, Aurora, USA

**Keywords:** 3d printed, anatomy, dissection, mastoidectomy, surgical education, temporal bone surgery

## Abstract

Introduction: Cadaveric temporal bones (CTBs) for otologic instruction have become increasingly expensive and scarce. Simulators offer a potentially cost-effective alternative to cadaveric specimens but must possess material characteristics similar to those of cadaveric specimens to be of value. This study evaluated otolaryngologists' interactions with additively manufactured (AM) analogs of cadaveric cortical and trabecular temporal bone and assessed how these interactions correlated with otologic experience.

Materials and methods: Solid and porous photopolymer samples with unique geometry profiles were created from multiple materials using a Stratasys J750 inkjet 3D printer (Stratasys Ltd., Rehovot, Israel). Surveys using a customized version of the Michigan Standard Simulation Scale Experience (MiSSES) were administered to otolaryngology trainees, otologists, and pediatric otolaryngologists to evaluate AM temporal bone (AMTB) analogs and a CTB. Kendall’s tau was used to compare Likert-scale responses to training year and level of otologic experience. Cronbach’s α for interrater reliability was calculated based on training. To account for multiple comparisons from correlations, p-values were adjusted using the Benjamini-Hochberg false discovery rate.

Results: Nineteen responses from thirteen otolaryngology trainees, two otologists, and four pediatric otolaryngologists with extensive otologic experience were included. All participants consistently rated both AMTB analogs positively for fidelity to cadaveric bone. Increasing surgical experience, as measured by both training year and completed mastoidectomies, was associated with higher survey scores for the AM analogs; however, after adjusting for multiple comparisons, there was no statistically significant association between training year or mastoidectomy experience and survey responses.

Discussion: Otologic training simulators must possess structural and material properties that closely match those of the human temporal bone. All respondents validated both solid and porous AM bony analogs, with a non-significant trend toward higher satisfaction with increasing surgical experience.

Conclusions: This study provides the basis for constructing CT-based AMTB models that provide high-fidelity haptic feedback for otologic training.

## Introduction

The acquisition of otologic surgical skills by otolaryngology trainees has traditionally involved mentored practice of complex surgical procedures in cadaveric temporal bone (CTB) specimens. Recently, this standard approach has been challenged by significant limitations in CTB availability. As CTBs become increasingly scarce and expensive to obtain, concerns about infection risk have also grown [1-3]. Although CTBs are considered the gold standard for surgical education, there is also limited access to temporal bones with pathology that otolaryngologists treat during these procedures. These limitations have highlighted the need for validated, high-fidelity temporal bone simulators to supplement otologic surgical training.

Additively manufactured (3D-printed) temporal bone (AMTB) simulators are receiving increasing attention as an alternative to cadaveric tissue for otology training [1-5]. The use of surgical simulators is valuable for otolaryngology trainees' acquisition of skill sets transferable to the operating room [6-7]. In addition to cost reductions resulting from advances in additive manufacturing (AM) technology, significant improvements in printer capabilities and materials have greatly increased the sophistication and fidelity of simulators used in medical training [8,9].

The literature reports numerous efforts to identify materials that can be processed by AM techniques to create high-fidelity AMTB simulators for otologic training [10-13]. Differences in material elasticity and melting temperature have led to variable results regarding haptic feedback and surgeons' experiences [10,13].

Validating material characteristics similar to those of cortical and trabecular temporal bone for use in AMTB simulators would be a step toward achieving realistic models for otologic training. Many studies evaluating different materials for AMTB models have used the Michigan Standard Simulation Scale Experience on a Likert scale. The objective of this study was to evaluate AM analogs of cadaveric cortical and cancellous bone composed of a novel UV-cured photopolymer to provide high-fidelity surgeon interactions compared with temporal bone components commonly encountered during mastoidectomy. We hypothesized that this novel UV-cured photopolymer, fabricated as solid and porous AM bony analogs, could provide a high-fidelity simulation of cortical and intramastoid trabecular temporal bone.

## Materials and methods

Solid and porous photopolymer samples with unique geometry profiles were created from multiple materials using a Stratasys J750 inkjet 3D printer (Stratasys Ltd., Rehovot, Israel). The J750 is a multimaterial inkjet 3D printer that deposits up to seven materials in a single print and can create digital mixtures of these materials across various geometries, including non-curing liquid materials within the print area [14,15]. The printer has an XYZ resolution of 80 × 40 × 27 µm (voxel sizes). These samples were developed from data acquired with high-definition CT of the temporal bone at 0.625-mm resolution. Geometric data were extracted from DICOM files of anonymous high-resolution CT scans of the temporal bone and processed using OpenVCAD (Matter Assembly Computation Lab, University of Colorado Boulder, Boulder, CO, USA). OpenVCAD is a multimaterial implicit volumetric modeling environment that samples from implicit volumes at the final step of output compilation. This approach preserves model accuracy while leveraging OpenCL for GPU-accelerated, high-performance generation of 30+ billion voxel models. Additional 3D image-processing steps based on a volumetric convolutional kernel were used to reduce high-frequency image noise or apply blending across individual material channels, as required for each model. A slicing algorithm was used to generate a stack of PNG slices from the volumetric representation as the final step of the compilation output. These PNG slices were used as input for the multimaterial inkjet 3D printer.

**Figure 1:**
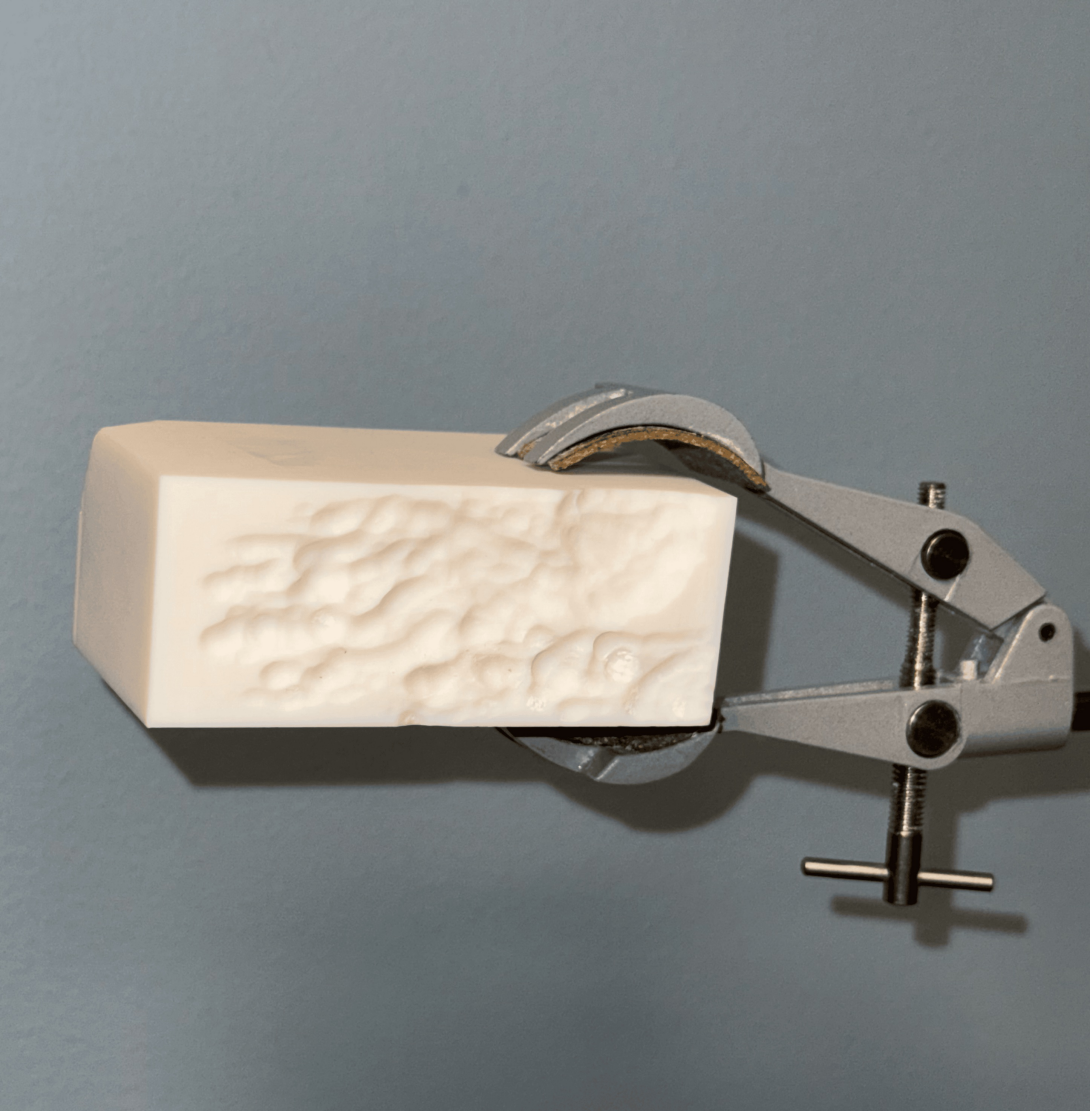
Cortical bony configuration of the AMTB analog AMTB: additively manufactured temporal bone

**Figure 2:**
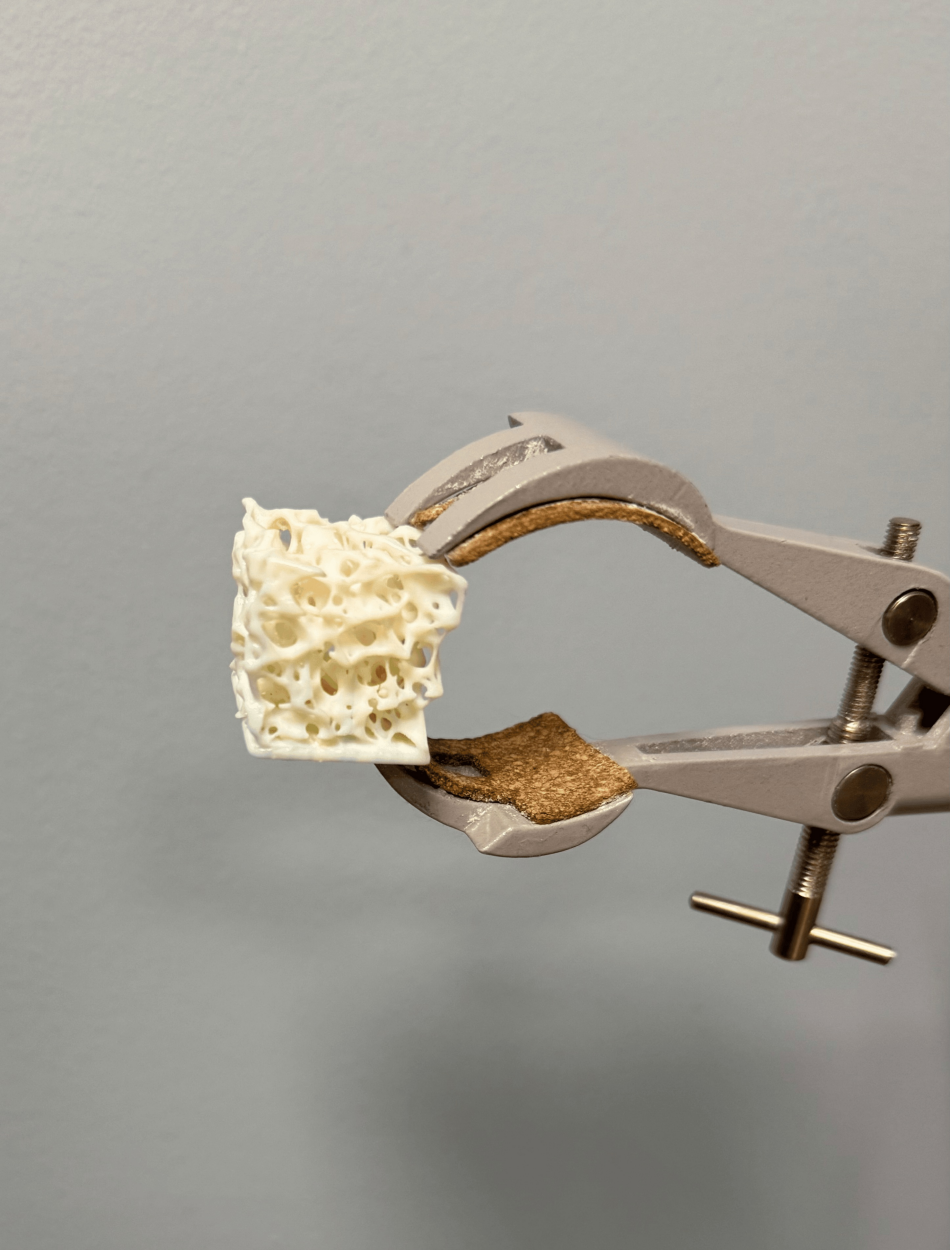
Trabecular (cancellous) bony configuration of the AMTB analog AMTB: additively manufactured temporal bone

**Figure 3:**
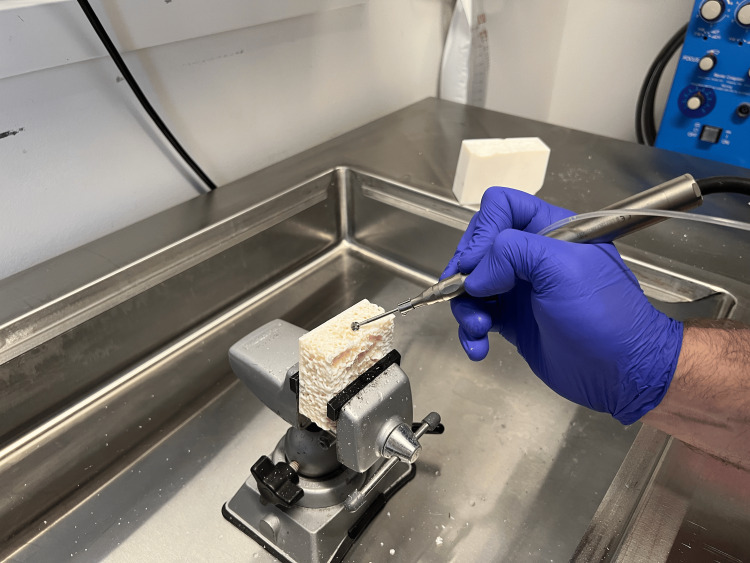
Laboratory setup for drilling the AMTB analog AMTB: additively manufactured temporal bone

The bony analogs (Figures 1-3, Table 1) were constructed using VeroWhite, a photopolymer cured with UV light, to provide properties similar to those of human cadaveric cortical and trabecular mastoid bone. The J750 prints with proprietary photo-acrylate materials; the manufacturer has not published a complete chemical formulation, but prior studies have conducted quasi-static load testing of the VeroWhite material used in this work [16,17].

**Table 1:** Materials utilized in AMTB simulators Fidelity: material performance compared to cadaveric bone, haptics: haptic feedback of material compared to cadaveric bone, FDM: fused deposition modeling, AMTB: additively manufactured temporal bone

Material	Printer	Model cost	Criteria	Likert scale parameters	Likert score	Source
Thermoplastic polycarbonate	Fortus	$14	Fidelity	1-dissimilar, 4-similar	2.71	[10]
Acrylonitrile butadiene styrene	Markerbot	$2	Fidelity	1-dissimilar, 4-similar	2.5	[10]
White resin	Objet	$25	Fidelity to cadaveric bone	1-dissimilar, 4-similar	2.75	[10]
Photopolymer	Formlabs	$5	Fidelity	1-dissimilar, 4-similar	2.64	[10]
White polylactic acid	FDM printer	$0.90	Appearance, haptics	1-dissimilar, 10-similar	Haptics 7.4, appearance 7.6	[12]
White acrylonitrile butadiene styrene	FDM printer	$0.90	Appearance, haptics	1-dissimilar, 10-similar	Haptics 7.1, appearance 7.2	[12]
White nylon	FDM printer	$0.90	Appearance, haptics	1-dissimilar, 10-similar	Haptics 5.6, appearance 6.7	[12]
Polyethylene terephthalate	FDM printer	Not reported	Appearance, haptics	1-dissimilar, 10-similar	Haptics 8.3, appearance 7.6	[12]
Natural polycarbonate	FDM printer	Not reported	Appearance, haptics	1-dissimilar, 10-similar	Haptics 7.4, appearance 3.9	[12]

Fourteen otolaryngology trainees from the University of Colorado Otolaryngology training program, along with two otology attendings and three pediatric otolaryngology attendings with extensive otology experience, participated in this study. Immediately after completing a temporal bone dissection course, participants were asked to perform drilling maneuvers using cutting and diamond burrs on both AMTB bony analogs in a simulated mastoidectomy procedure. Participants were then asked to complete an anonymous survey of their experience with the cortical and trabecular AMTB analogs. This study was approved by the Colorado Multiple Institutional Review Board.

Surveys employed a customized version of the Michigan Standard Simulation Scale Experience (MiSSES), consisting of 23 questions grouped into three domains: material interactions with the otologic drill, educational value, and overall model quality relative to cadaveric bone (Figure 1) [18]. The aspects used from the instrument were model fidelity and educational value. The adapted survey used in this study has not been previously validated. Responses were scored using a 5-point Likert scale ranging from (1) strongly disagree to (5) strongly agree. A score of 3 suggested that the AMTB analog was an acceptable alternative to cadaveric bone. Scores below 3 indicated poor similarity between the AMTB analog and cadaveric bone, whereas scores above 4 implied high fidelity of the AMTB material to cadaveric bone.

Descriptive statistics are reported as frequency (%). Internal consistency was assessed by training level (otolaryngology trainees and otology/pediatric otolaryngology attending groups) using Cronbach’s α. Kendall’s correlations were used to associate Likert-scale responses with training year and number of mastoidectomy procedures performed, with correlation coefficients (Kendall’s tau, τ) and p-values reported. A positive correlation coefficient indicates that as training year or number of mastoidectomies increases, agreement with the given survey question also increases. A negative correlation coefficient indicates that as training year or number of mastoidectomy procedures performed increases, agreement with the given survey question decreases. To account for multiple comparisons from correlations, p-values were adjusted using the Benjamini-Hochberg false discovery rate. P-values < 0.05 were considered statistically significant. All analyses were performed in R version 4.3.2 (R Foundation for Statistical Computing, Vienna, Austria).

## Results

There were 19 survey responses from 13 otolaryngology trainees, two otologists, and four pediatric otolaryngologists with extensive otologic experience (Table 2).

**Table 2:** Respondent demographics N represents the number and percentage of each category out of the total number of respondents. Percentage out of 19 total respondents. PGY: postgraduate year, ENT: ear, nose, and throat

Training year	Overall (N = 19)
4th year medical student	1 (5.26%)
Attending	5 (26.3%)
Fellow	1 (5.26%)
PGY 2	3 (15.8%)
PGY 3	5 (26.3%)
PGY 4	2 (10.5%)
PGY 5	2 (10.5%)
Specialty	
ENT	1 (5.26%)
ENT resident	12 (63.2%)
Otology	2 (10.5%)
Ped ENT	4 (21.1%)
Number of mastoidectomy procedures performed	
<10	9 (47.4%)
11-25	3 (15.8%)
51-100	2 (10.5%)
101-500	1 (5.26%)
>500	4 (21.1%)

Percentages were calculated based on 19 respondents. The number of mastoidectomies performed by respondents ranged from fewer than 10 (nine participants) to more than 500 (four participants). All AMTB analogs were consistently rated positively for fidelity relative to their cadaveric bony counterparts (Table 3).

**Table 3:** Survey responses Survey responses were reported as median, Q1, Q3, minimum, and maximum (1 = strongly disagree/dislike, 2 = somewhat disagree/dislike, 3 = neutral, 4 = somewhat agree/like, 5 = strongly agree/like). N(%) represents the percentage of each category out of the total number of respondents.

	Likert scale					Median (Q1, Q3)			
	1	2	3	4	5		Overall	Attending/fellow	Trainee
The material used has adequately realistic characteristics/features of a bone sample	0 (0%)	0 (0%)	0 (0%)	7 (36.8%)	12 (63.2%)		5 (4, 5)	5 (5, 5)	4 (4, 5)
Drilling the solid sample produces a similar sound to cortical bone	0 (0%)	2 (11.8%)	1 (5.9%)	6 (35.3%)	8 (47.1%)		4 (4, 5)	4.5 (3.5, 5)	4 (4, 5)
Drilling the solid sample produces similar tactile feedback to cortical bone	0 (0%)	1 (5.3%)	2 (10.5%)	5 (26.3%)	11 (57.9%)		5 (4, 5)	5 (5, 5)	4 (4, 5)
The solid sample was harder than cortical bone	9 (47.4%)	4 (21.1%)	1 (5.3%)	3 (15.8%)	2 (10.5%)		2 (1, 3.5)	1 (1, 2.5)	2 (1, 4)
The solid sample was softer than cortical bone	10 (52.6%)	4 (21.1%)	1 (5.3%)	4 (21.1%)	0 (0%)		1 (1, 2.5)	1.5 (1, 2.75)	1 (1, 2)
The drill passes through the solid sample similar to cortical bone	0 (0%)	0 (0%)	2 (10.5%)	5 (26.3%)	12 (63.2%)		5 (4, 5)	5 (4.25, 5)	5 (4, 5)
The drilled material (fragments) was similar to bone dust of cortical bone	0 (0%)	5 (27.8%)	0 (0%)	4 (22.2%)	9 (50%)		4.5 (2.5, 5)	5 (5, 5)	4 (2, 5)
Inadvertent drill bit digging into material (chatter) of solid sample was similar to cortical bone	0 (0%)	1 (5.9%)	1 (5.9%)	6 (35.3%)	9 (52.9%)		5 (4, 5)	5 (4.75, 5)	4 (4, 5)
Drilling the porous sample produces a similar sound to trabecular bone	0 (0%)	2 (11.1%)	0 (0%)	8 (44.4%)	8 (44.4%)		4 (4, 5)	5 (5, 5)	4 (4, 4)
Drilling the porous sample produces a similar tactile feedback to trabecular bone	0 (0%)	0 (0%)	0 (0%)	11 (57.9%)	8 (42.1%)		4 (4, 5)	5 (4.25, 5)	4 (4, 5)
The porous sample was harder than trabecular bone	9 (47.4%)	2 (10.5%)	3 (15.8%)	4 (21.1%)	1 (5.3%)		2 (1, 3.5)	1.5 (1, 2.75)	2 (1, 4)
The porous sample was softer than trabecular bone	12 (66.7%)	1 (5.6%)	3 (16.7%)	2 (11.1%)	0 (0%)		1 (1, 2.75)	1 (1, 3)	1 (1, 2)
The drill passes through the porous sample similar to trabecular bone	1 (5.3%)	0 (0%)	1 (5.3%)	9 (47.4%)	8 (42.1%)		4 (4, 5)	5 (4.25, 5)	4 (4, 5)
The drilled material (fragments) was similar to bone dust of trabecular bone	1 (5.6%)	1 (5.6%)	2 (11.1%)	4 (22.2%)	10 (55.6%)		5 (4, 5)	5 (5, 5)	4 (3, 5)
Inadvertent drill bit digging into material (chatter) of solid sample was similar to trabecular bone	0 (0%)	1 (5.6%)	2 (11.1%)	9 (50%)	6 (33.3%)		4 (4, 5)	4 (4, 5)	4 (4, 5)
The solid material did not melt with diamond drill bit use	1 (5.9%)	0 (0%)	0 (0%)	2 (11.8%)	14 (82.4%)		5 (5, 5)	5 (5, 5)	5 (4.75, 5)
The porous material did not melt with diamond drill bit use	1 (5.6%)	0 (0%)	0 (0%)	2 (11.1%)	15 (83.3%)		5 (5, 5)	5 (5, 5)	5 (5, 5)
Suction irrigator handling of debris was similar to cadaveric bone	0 (0%)	0 (0%)	0 (0%)	2 (14.3%)	12 (85.7%)		5 (5, 5)	5 (5, 5)	5 (5, 5)
Suction irrigator obstruction frequency was similar to cadaveric bone	0 (0%)	0 (0%)	2 (16.7%)	2 (16.7%)	8 (66.7%)		5 (4, 5)	5 (5, 5)	5 (4, 5)
A temporal bone model made of this material may serve as a useful temporal bone simulator	0 (0%)	0 (0%)	0 (0%)	4 (21.1%)	15 (79%)		5 (5, 5)	5 (5, 5)	5 (5, 5)
Drilling on a temporal bone model made of this material would provide similar feedback (how the drill feels in the surgeon’s hand) to cadaveric temporal bone	0 (0%)	1 (5.3%)	0 (0%)	6 (31.6%)	12 (63.2%)		5 (4, 5)	5 (4.25, 5)	5 (4, 5)
Use of temporal bone model with this material would serve as a useful adjunct to cadaveric temporal bone lab work	0 (0%)	0 (0%)	0 (0%)	3 (15.8%)	16 (84.2%)		5 (5, 5)	5 (5, 5)	5 (5, 5)
Overall similarity of solid material compared to cortical bone	0 (0%)	0 (0%)	1 (5.3%)	7 (36.8%)	11 (57.9%)		5 (4, 5)	5 (5, 5)	4 (4, 5)
Overall similarity of porous material compared to trabecular bone	0 (0%)	0 (0%)	1 (5.3%)	6 (31.6%)	12 (63.2%)		5 (4, 5)	5 (5, 5)	5 (4, 5)
Overall rating of material	0 (0%)	0 (0%)	0 (0%)	6 (31.6%)	13 (68.4%)		5 (4, 5)	5 (5, 5)	5 (4, 5)

Ratings for educational value, material interactions with the otologic drill, and overall model quality were also favorable. All respondents deemed the material in both configurations to be suitable analogs for use in a future AMTB simulator. Responses did not differ significantly by either training year or prior mastoidectomy experience.

Survey responses indicated that the AMTB material demonstrated excellent fidelity to both cortical and trabecular bone, with higher satisfaction levels associated with greater otologic experience (Table 3). Both the cortical and trabecular AMTB analogs were highly rated, with median scores of 4 or higher across fidelity, haptic feedback, educational value, and overall quality/responsiveness measures. No melting of the material during diamond burr use was observed. Kendall’s tau was used to assess the association between Likert-scale responses and training year and the number of mastoidectomy procedures performed (Tables 4-5).

**Table 4:** Kendall’s correlations between survey responses and training year p < 0.05 is considered significant. Values denoted with "*" are considered significant values. There were no correlations that remained statistically significant after adjustment for false discovery rate.

	Correlation coefficient	p-value	Adjusted p-value
The material used has adequately realistic characteristics/features of a bone sample	0.26	0.25	0.48
Drilling the solid sample produces a similar sound to cortical bone	-0.03	0.92	1
Drilling the solid sample produces similar tactile feedback to cortical bone	0.21	0.34	0.59
The solid sample was harder than cortical bone	-0.34	0.1	0.48
The solid sample was softer than cortical bone	-0.08	0.73	0.92
The drill passes through the solid sample similar to cortical bone	0.1	0.65	0.9
The drilled material (fragments) was similar to bone dust of cortical bone	0.55	0.01*	0.14
Inadvertent drill bit digging into material (chatter) of solid sample was similar to cortical bone	0.29	0.2	0.48
Drilling the porous sample produces a similar sound to trabecular bone	0.52	0.02*	0.14
Drilling the porous sample produces a similar tactile feedback to trabecular bone	0.44	0.05	0.28
The porous sample was harder than trabecular bone	-0.07	0.74	0.92
The porous sample was softer than trabecular bone	0.19	0.4	0.62
The drill passes through the porous sample similar to trabecular bone	0.32	0.15	0.48
The drilled material (fragments) was similar to bone dust of trabecular bone	0.61	< 0.01*	0.1
Inadvertent drill bit digging into material (chatter) of solid sample was similar to trabecular bone	0.17	0.45	0.66
The solid material did not melt with diamond drill bit use	0.29	0.22	0.48
The porous material did not melt with diamond drill bit use	0.27	0.25	0.48
Suction irrigator handling of debris was similar to cadaveric bone	0.34	0.22	0.48
Suction irrigator obstruction frequency was similar to cadaveric bone	0.28	0.36	0.59
A temporal bone model made of this material may serve as a useful temporal bone simulator	-0.07	0.79	0.92
Drilling on a temporal bone model made of this material would provide similar feedback (how the drill feels in the surgeon’s hand) to cadaveric temporal bone	-0.06	0.81	0.92
Use of temporal bone model with this material would serve as a useful adjunct to cadaveric temporal bone lab work	0	1	1
Overall similarity of solid material compared to cortical bone	0.34	0.12	0.48
Overall similarity of porous material compared to trabecular bone	0.3	0.17	0.48
Overall rating of material	0.01	1	1

**Table 5:** Kendall’s correlations between survey responses and number of mastoidectomy procedures performed p < 0.05 is considered significant. Values denoted with "*" are considered significant values. None of the Kendall's tau correlations were significant after adjusting for the false discovery rate.

	Correlation coefficient	p-value	Adjusted p-value
The material used has adequately realistic characteristics/features compared of a bone sample	0.4	0.07	0.35
Drilling the solid sample produces a similar sound to cortical bone	0.1	0.7	0.91
Drilling the solid sample produces similar tactile feedback to cortical bone	0.35	0.1	0.35
The solid sample was harder than cortical bone	-0.29	0.16	0.45
The solid sample was softer than cortical bone	0.08	0.74	0.93
The drill passes through the solid sample similar to cortical bone	0.16	0.48	0.72
The drilled material (fragments) was similar to bone dust of cortical bone	0.52	0.02*	0.2
Inadvertent drill bit digging into material (chatter) of solid sample was similar to cortical bone	0.29	0.21	0.52
Drilling the porous sample produces a similar sound to trabecular bone	0.54	0.01*	0.2
Drilling the porous sample produces a similar tactile feedback to trabecular bone	0.37	0.09	0.35
The porous sample was harder than trabecular bone	0.06	0.78	0.93
The porous sample was softer than trabecular bone	0.23	0.3	0.63
The drill passes through the porous sample similar to trabecular bone	0.25	0.25	0.56
The drilled material (fragments) was similar to bone dust of trabecular bone	0.41	0.06	0.35
Inadvertent drill bit digging into material (chatter) of solid sample was similar to trabecular bone	0.16	0.47	0.72
The solid material did not melt with diamond drill bit use	0.18	0.46	0.72
The porous material did not melt with diamond drill bit use	0.15	0.53	0.73
Suction irrigator handling of debris was similar to cadaveric bone	0.05	0.92	1
Suction irrigator obstruction frequency was similar to cadaveric bone	0.41	0.15	0.45
A temporal bone model made of this material may serve as a useful temporal bone simulator	-0.16	0.49	0.72
Drilling on a temporal bone model made of this material would provide similar feedback (how the drill feels in the surgeon’s hand) to cadaveric temporal bone	-0.01	1	1
Use of temporal bone model with this material would serve as a useful adjunct to cadaveric temporal bone lab work	0.01	1	1
Overall similarity of solid material compared to cortical bone	0.46	0.03*	0.28
Overall similarity of porous material compared to trabecular bone	0.18	0.42	0.72
Overall rating of material	-0.06	0.82	0.93

None of the p-values remained statistically significant after adjustment for the false discovery rate. However, most survey responses demonstrated positive correlation coefficients, indicating that agreement with the survey questions tended to increase with both training year and number of mastoidectomy procedures performed.

The Cronbach’s α values of 0.85 (95% CI: 0.59-0.98) and 0.82 (95% CI: 0.63-0.93) indicate high internal consistency of the survey among the otolaryngology trainee group and the otology/pediatric otolaryngology attending group, respectively (Table 6).

**Table 6:** Cronbach’s α PGY: postgraduate year, CI: confidence interval

Training	Cronbach’s α (95% CI)	
Attending/fellow	0.85 (0.59, 0.98)	
PGY 2-5/4th year medical student	0.82 (0.63, 0.93)	

All respondents agreed that an AMTB model composed of this bony analog would serve as an excellent educational tool for otologic training.

## Discussion

Interest in simulation for surgical education has steadily increased over the last 20 years, with numerous studies demonstrating its value in acquiring surgical skills [9]. In otolaryngology, the decreased availability of CTBs has prompted investigations into AMTB models for otologic training [1-5]. In addition to eliminating potential infectious vectors associated with cadaveric specimens, AMTB simulators can provide relatively low-cost, on-demand access to disease-specific or patient-specific conditions [8,9]. A meta-analysis found that AM interventions in otolaryngology demonstrated surgical, anatomical, and educational value [19]. Omari et al.'s systematic review also found promise in improving surgical precision and reducing operative times with patient-specific AMTB models; however, it recommended additional studies to evaluate how well these translated into patient outcomes [20]. AMTB simulators have been validated as anatomically accurate and useful training models for otologic procedures such as mastoidectomy [21,22]. Hong et al.'s systematic review found AMTB models highly valuable as teaching tools and predicted their increasing integration into otolaryngology training [23].

It is accepted that AMTB simulators must possess anatomical and material characteristics that closely match those of the CTB. While current temporal bone models rely on complex, image-guided 3D reconstructions [24,25] to achieve anatomic fidelity, identifying AM materials that are representative of bone remains an active area of investigation. While Meglioli et al. agreed with the surgical and educational value of patient-specific AMTB models, they also noted that the diversity of printing technologies and materials requires clinical users to adapt the technology to their specific needs [26]. Mowry et al. found that stereolithography printers with photocrosslinkable white acrylic resin (FLW) were superior to polycarbonate, gray resin, Visijet PXL calcium sulfate hemihydrate, wood/PLA filament, and resin [13]. Haffner et al. compared five different AMTB materials, finding that polyethylene terephthalate most closely resembled temporal bone, whereas acrylonitrile butadiene styrene, polycarbonate, and nylon showed poor correlation [12]. McMillen et al. noted the best drilling experience with FLW but found better anatomic performance with photopolymer and polycarbonate materials [10]. Multimaterial AMTB models have also been developed to represent soft and bony tissues, as well as cholesteatoma [1]. Many AM materials used in AMTB models have been studied and found to comply with occupational hazard standards [27].

The high costs of printers and materials hampered prior attempts to create high-fidelity AMTB models comparable to CTBs. As technology has improved over the last 10-15 years, costs have also significantly decreased, with recent reports indicating that raw materials cost roughly $10 per model after the initial investment in all required equipment [28]. When comparing the costs of an AMTB model and a CTB, the literature quotes CTBs at roughly $300-$500, with limited supply often posing a significant barrier to acquisition [29]. Multiple studies have shown that AMTB models closely resemble the anatomy and feel of real temporal bone during dissection; however, prior groups cited the high upfront costs of printers and materials as prohibitive to widespread application [29]. Multimaterial PolyJet inkjet technology can produce models containing up to seven materials, with elastic moduli ranging from 30 kPa to 2 GPa. PolyJet 3D printing has a relatively high throughput of 1 liter per hour, and each liter of material costs approximately $500 [14]. The material used for our model costs roughly $850/3.7 kg, or $0.23/g. With approximately 40 mL of resin per model, this amounts to about $3 per model, which is consistent with the literature [29,30]. As the costs of this technology have progressively decreased, advances in AM have created an opportunity to meet the need for high-volume simulation in residency training.

Cost, availability, and safety are important advantages of AMTB models; however, the importance of feel and anatomic accuracy should not be overlooked when compared with CTB dissections. It should be noted that this study was conducted in a temporal bone laboratory with standard ventilation but was not performed in a laboratory hood. All participants wore gloves and standard masks; however, no particulate measurements were taken during this study, representing an important area for future investigation as AMTB models become more widely used for resident education. Participants’ evaluations of this material in terms of its material characteristics relative to bone, tactile feedback, and overall experience were positive and consistent with the literature demonstrating the value of AMTB models across various materials [1,7,10]. When study responses were correlated with experience, greater satisfaction with the bony analogs was observed for both cortical and trabecular configurations.

This pilot study demonstrated the feasibility of this novel material for use in an AMTB model, with haptic feedback similar to that of cadaveric bone, as noted by study participants regardless of training level or degree of mastoidectomy experience. This study also demonstrated that the material can realistically simulate both cortical and trabecular bone configurations. Given these results, the next phases of our investigation will validate an anatomically complete AMTB model composed of this novel material and conduct further testing to ensure its safety for use. This will be followed by the development of a more sophisticated multiphase composite AMTB simulator containing both soft and hard tissues, which is the focus of the RM laboratory. Through multimaterial additive manufacturing, these models can replicate complex structures ranging from liquid to bone and incorporate color and texture into individual components. Multiphase AMTB simulators also enable modeling of disease states such as cholesteatoma, vascular tumors, and mastoiditis, providing trainees with disease-specific simulations that CTBs cannot replicate.

As with most evaluations of surgical training simulators, this single-institution study was limited by a relatively small sample size and the use of a non-validated version of the MiSSES survey. There was an imbalance between trainees and attendings, with potential response bias given that all participants had just completed a cadaveric dissection. There was no comparison with other commonly used materials for AMTB models or with complete AMTB models. Responses from study participants were subjective, and there was no direct comparison with other materials previously used in AMTB models. While participants generally rated the material favorably, we did not observe statistically significant associations with training year or number of mastoidectomies performed after adjustment for multiple comparisons; however, most correlation coefficients were positive, suggesting greater agreement with survey questions among more experienced participants. While this could indicate that the model would perform well as a training tool, further investigation using a complete AMTB model for skill-based tasks would strengthen this conclusion. Applicability is difficult to assess in this small, single-institution study involving a novel material; comparisons with other AM materials and evaluation using a complete AMTB model are needed. The use of this material as a bony analog in a multimaterial AMTB model is also a future goal beyond the scope of this pilot study.

## Conclusions

This study demonstrated that AMTB bony analogs resembling cortical and trabecular bone, similar to cadaveric mastoid bone, can be synthesized, suggesting that these materials can be incorporated into a realistic AMTB model for otologic training. The bony analogs evaluated in this study were generally perceived as high-fidelity by this small sample of participants. Our preliminary data support further development and a more robust investigation into whether incorporating this material into a complete AMTB model can provide a high-quality educational tool that supplements or replaces cadaveric specimens. As AM technology continues to advance and cadaveric specimens become increasingly scarce, AMTB models will likely become an increasingly important adjunct to surgical training. This material’s ability to provide a high-fidelity surgical experience comparable to CTB specimens, as well as its potential for incorporation into future multiphase composite AMTB simulators that replicate various otologic disease states, warrant further investigation.
